# Biosignatures Associated with Freshwater Microbialites

**DOI:** 10.3390/life10050066

**Published:** 2020-05-15

**Authors:** Richard Allen White, Sarah A. Soles, Allyson L. Brady, Gordon Southam, Darlene S.S. Lim, Greg F. Slater

**Affiliations:** 1 Department of Plant Pathology, Washington State University, Pullman, WA 99163, USA; raw937@gmail.com; 2 RAW Molecular Systems (RMS) LLC, Spokane, WA 99218, USA; 3 Australian Centre for Astrobiology, University of New South Wales, Sydney, NSW 2052, Australia; 4 School of Geography and Earth Sciences, McMaster University, Hamilton, ON L8S 4K1, Canada; sarah.a.soles@gmail.com (S.A.S.); bradyal@mcmaster.ca (A.L.B.); 5 School of Earth and Environmental Sciences, University of Queensland, QLD 4072, Australia; g.southam@uq.edu.au; 6 NASA Ames Research Center, Moffett Field, CA 94035, USA; Darlene.Lim@nasa.gov

**Keywords:** Kelly Lake, Pavilion Lake, microbialites, biosignatures

## Abstract

Freshwater microbialites (i.e., lithifying microbial mats) are quite rare in northern latitudes of the North American continent, with two lakes (Pavilion and Kelly Lakes) of southeastern BC containing a morphological variety of such structures. We investigated Kelly Lake microbialites using carbon isotope systematics, phospholipid fatty acids (PLFAs) and quantitative PCR to obtain biosignatures associated with microbial metabolism. δ^13^C_DIC_ values (mean δ^13^C_DIC_ −4.9 ± 1.1‰, *n* = 8) were not in isotopic equilibrium with the atmosphere; however, they do indicate ^13^C-depleted inorganic carbon into Kelly Lake. The values of carbonates on microbialite surfaces (δ^13^C) fell within the range predicted for equilibrium precipitation from ambient lake water δ^13^C_DIC_ (−2.2 to −5.3‰). Deep microbialites (26 m) had an enriched δ^13^C_carb_ value of −0.3 ± 0.5‰, which is a signature of photoautotrophy. The deeper microbialites (>20 m) had higher biomass estimates (via PLFAs), and a greater relative abundance of cyanobacteria (measured by 16S copies via qPCR). The majority of PLFAs constituted monounsaturated and saturated PLFAs, which is consistent with gram-negative bacteria, including cyanobacteria. The central PLFA δ^13^C values were highly depleted (−9.3 to −15.7‰) relative to δ^13^C values of bulk organic matter, suggesting a predominance of photoautotrophy. A heterotrophic signature was also detected via the depleted *iso-* and *anteiso-*15:0 lipids (−3.2 to −5.2‰). Based on our carbonate isotopic biosignatures, PLFA, and qPCR measurements, photoautotrophy is enriched in the microbialites of Kelly Lake. This photoautotrophy enrichment is consistent with the microbialites of neighboring Pavilion Lake. This indication of photoautotrophy within Kelly Lake at its deepest depths raises new insights into the limits of measurable carbonate isotopic biosignatures under light and nutrient limitations.

## 1. Introduction

Understanding the processes involved in microbialite carbonate precipitation is important for interpreting the geological record and recognizing any potential biosignatures that are preserved. The significance of photoautotrophic versus heterotrophic organisms in microbialite building has been shown to vary across field sites such as Highbourne Cay, Bahamas [1], Cuatro Ciénegas, Mexico [2,3], Shark Bay, Australia [4], Lake Van [5], Satonda crater lake [6], Clinton Creek [7] and Pavilion Lake, Canada [8,9]. Photoautotrophic processes, particularly carbon fixation by cyanobacteria, have been demonstrated to play a role in the precipitation of calcium carbonate in a number of environments [10,11,12,13,14].

During oxygenic photosynthesis in an aqueous setting (pH~7–8), dissolved HCO_3_^−^ is intracellularly converted to CO_2_ and OH^−^ by the actions of carbonic anhydrase [15]. While the CO_2_ is incorporated into organic biomass, the OH^−^ is excreted from the cell and results in an increase in the surrounding pH. This change in pH leads to a shift in the dissolved inorganic carbon (DIC) speciation and, in turn, increases the saturation index of calcium carbonate that can result in mineral precipitation [8,14,16,17,18]. Photoautotrophic processes can also alter the carbon isotopic composition of the local DIC through the preferential uptake and conversion of ^12^CO_2_ into organic material, with ^13^C-enrichement of the residual DIC, an effect that can subsequently be preserved as an isotopic biosignature in the δ^13^C values of the carbonate that precipitates [8,19,20,21,22,23,24]. Heterotrophic organisms can also influence calcium carbonate precipitation via the oxidation of ^13^C-depleted organic carbon, which increases the concentration of total DIC [16,25,26]. When carbonate precipitates under these conditions, it can result in a heterotrophic biosignature with a δ^13^C_carb_ value more negative than that produced under equilibrium conditions [2,22].

Beyond the carbonate isotopic effects, the presence of autotrophs and/or heterotrophs in a microbial community can also be examined through the carbon isotopic composition of the phospholipid fatty acids (PLFAs) relative to that of the bulk organic carbon. PLFAs are components of bacterial and eukaryotic cell membranes which have been shown to degrade rapidly upon cell death [27]. Therefore, they are useful indicators of the biomass and, in some cases, provide the general composition of the viable microbial community [28,29,30]. The δ^13^C values of PLFAs are dependent on the isotopic composition of the carbon source, as well as the metabolisms of the microbes that synthesize them [31,32]. Aerobic heterotrophs tend to produce PLFAs depleted by 0–4‰ relative to the bulk organic material δ^13^C value [30,33], whereas autotrophs such as cyanobacteria synthesize PLFAs depleted by 7–13‰ relative to the bulk cell [34].

Pavilion Lake, located in British Columbia, Canada, is an example of a site where photoautotrophic metabolisms have been shown to be an important factor in microbialite formation [8,9,35,36]. Kelly Lake is located approximately 20 km from Pavilion Lake and also hosts large meter-scale microbialites in addition to smaller thrombolites [20,37]. While both Pavilion and Kelly Lake host microbialites, other lakes in the region do not [37], however the reasons for this are not entirely clear. Despite the geographic similarity, differences exist between Pavilion and Kelly that may influence the growth mechanisms and morphology of the microbialites, offering insight into the formation of these structures throughout the geologic record. Some of the differences that exist within Kelly Lake compared to Pavilion include: (1) lack of thick, cohesive surface mats, (2) higher levels of arthropods (e.g., mainly insects) and (3) the ‘degraded’ appearance of the microbialites. It has been suggested that Kelly Lake microbialites, unlike those in Pavilion, are no longer actively growing [37]. Kelly Lake therefore represents a unique opportunity to test for biosignatures relating to microbial metabolisms that may differ from those of photosynthesis identified within Pavilion Lake [8,35]. The proximity of these two systems presents an opportunity to compare microbialite formation mechanisms between distinct, but co-located, sites. Our investigation examined the isotopic systematics, microbial community composition and biomarkers associated with freshwater microbialites located at varying depths in Kelly Lake. The questions related to this study are: (1) Are biosignatures detectable within Kelly Lake microbialites? and (2) Does the biotic signature (whether photoautotrophic or other) differ between Pavilion and Kelly lake? We hypothesize that Kelly Lake has actively growing microbialites, despite evidence for a degraded appearance and a relatively thin surface microbial mat, that show evidence of a photoautotrophic isotopic biosignature.

## 2. Methods

### 2.1. Site Description

**Figure 1:**
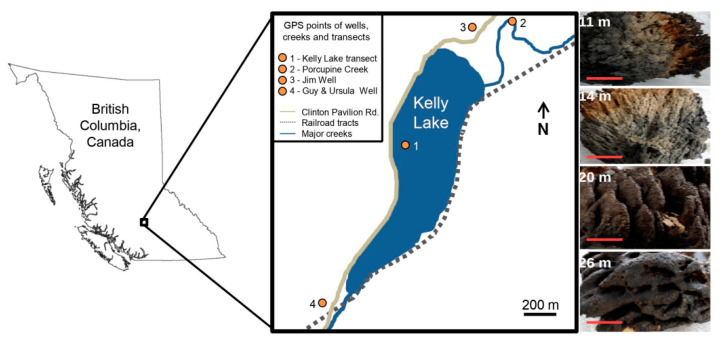
The location of Kelly Lake and the transect along which microbialites were collected. The wells and Porcupine Creek from which water samples were taken are also shown. The four representative Kelly Lake microbialites samples from depths (11, 14, 20, 26 m) are listed on the right with the red scale bar (10 cm).

Kelly Lake is located between the Edge Hills and Tsilsalt Ridge of the Marble Mountains, British Columbia, Canada, at an elevation of 1068 m [20]. A comprehensive limnological overview of Kelly Lake and the region surrounding this site was published by Lim et al. [37]. The following is a summary of specific data from this and other relevant studies for Kelly Lake [20,38]. Our study site is an alkaline (pH 8.3), ultra-oligotrophic freshwater lake with a maximum depth of ~40 m. The water is slightly supersaturated with respect to calcite and develops a seasonal thermocline between 5 and 15 m deep. DIC concentrations range from 36.5 mg L^−1^ at the surface and 37.8 mg L^−1^ at 30 m. The Pavilion Lake Research Project (PLRP) found large, meter-scale microbialites in Kelly Lake during their 2004 field season, thus providing an opportunity to conduct a comparative study in microbialite formation between Kelly and Pavilion lakes. Unlike Pavilion Lake, there is a distinct surface water inflow, Porcupine Creek, which enters the lake near the northern shore. An outflow stream towards Pear Lake is found at the southern tip of Kelly Lake (Figure 1).

### 2.2. Physical Properties

Vertical profiles of water temperature and electrical conductance were measured at the approximate center of Kelly Lake using SeaBird SBE19 and SBE19plus conductivity–temperature–depth (CTD) profilers. Specific conductance was estimated using the method of Pawlowicz [39] from measured in situ pressure, temperature, electrical conductance measurements and ionic content of water samples. Profiles of photosynthetically active radiation (PAR) were measured with a Licor Li-193 Underwater Spherical Quantum Sensor connected to a SeaBird SBE19plus profiler.

### 2.3. Sample Collection and Analysis

#### 2.3.1. Water Samples

Surface- and deep-water samples were collected for ^13^C analysis in crimp-sealed glass bottles with no headspace and fixed on site with mercuric chloride. Deep water samples were collected from 15 and 30 m using a Niskin water sampler (General Oceanics, Miami, FL, USA). The clarity of Kelly Lake, including a lack of observed whiting events, and analysis of materials collected from previous sediment traps suggest that CaCO_3_ derived from particulate material is a negligible constituent of the microbialites [37]. The total inorganic carbon in these samples is, therefore, assumed to be dissolved. The isotopic composition of the DIC was determined by acidification and analysis of the resultant CO_2_ using a continuous flow isotope ratio mass spectrometer at the G.G. Hatch Laboratory in Ottawa (St-Jean, 2003). All δ^13^C values are reported in standard delta notation in reference to PeeDee Belemnite (PDB). The sample collected for ^14^C analysis of surface water DIC was sent to the National Ocean Sciences Accelerator Mass Spectrometry Facility (NOSAMS) at Woods Hole Oceanographic Institute, where it was acidified and the resultant CO_2_ purified, then graphitized for analysis by accelerator mass spectrometry. The reported radiocarbon age was calibrated using OxCal version 4.1 (IntCal09) [40].

#### 2.3.2. Phospholipid Fatty Acid Extraction

Along a transect, three individual microbialites were collected by SCUBA divers from each depth of 11, 20, and 26 m (Figure 1). In addition, one microbialite was collected from 14 m and separated into three pieces. All samples were frozen at −20 °C on-site for transportation back to McMaster University. PLFAs were extracted from freeze-dried microbialite surface material using a modified Bligh and Dyer method [41], as described in Brady et al [35]. Briefly, dichloromethane (DMC), methanol, and a phosphate buffer solution were used in the ratio of 1:2:0.8 (*v*/*v*). The extract was filtered into a separatory funnel where DCM and water were added to achieve a MeOH/DCM/water mixture of 1:1:0.9. The lower organic phase was removed and silica gel liquid chromatography was used to separate the lipids in polar, neutral, and non-polar fractions. The polar fraction was subjected to mild alkaline methanolysis to convert any PLFAs into fatty acid methyl esters (FAMEs) [42], which were subsequently separated using gas chromatography mass spectrometry (GC/MS) on an Agilent GC/MS (Agilent Technologies Inc., Santa Clara, CA, USA) with an DB-5MS capillary column (30 m × 0.32 mm I.D. × 0.25 µm film thickness). This was done using a temperature program of 50 °C (1 min), 20 °C min^−1^ to 130 °C, 4 °C min^−1^ to 160 °C, 8° min^−1^ to 300 °C (5 min). PLFAs were identified by their retention times and mass spectra relative to those of known reference standards (Bacterial Acid Methyl Ester Mix, Matreya Inc., Pleasant Gap, PA, USA, and Supelco 37 Component FAME Mix, Sigma-Aldrich Co., Bellefonte, PA, USA). Reference standards were also used to identify double bond positions where possible. PLFAs are named according to their number of carbon atoms (a) and double bonds (b), using the notation a:b. Fatty acids with branches at unknown locations are denoted with the prefix ‘br’, whereas *iso-* and *anteiso-* branched PLFAs are identified by the prefixes ‘*i*’ and ‘*a*’, respectively. Cyclopropyl PLFAs are denoted by ‘cy’. Total PLFA concentrations are presented as means for each depth in µg PLFA/g of dry sample extracted ± one standard deviation (s.d.).

#### 2.3.3. Stable Isotope Analysis

Microbialite FAMEs were inserted using an injector set to the splitless mode at 300 °C and were subsequently separated using an Agilent GC/MS. An HP-88 capillary column (100 m × 0.25 mm I.D. × 0.2 µm film thickness) was used for all samples from 11 and 20 m, whereas a DB-5MS capillary column (30 m × 0.32 mm I.D. × 0.25 µm film thickness) was utilized for all 14 and 26 m samples. A temperature program of 80 °C (1 min), 10 °C min^−1^ to 175 °C (12 min), 2 °C min^−1^ to 190 °C (10 min), 10 °C min^−1^ to 240 °C (15 min) was applied to the HP-88 column, while a program of 80 °C (1 min), 4 °C min^−1^ to 280 °C, 10 °C min^−1^ to 320 °C (10 min) was applied to the DB-5 column. Individual FAMEs were eluted from the column and combusted in an oven set at 960 °C. The resultant CO_2_ was analyzed using a Delta^Plus^ XP continuous flow isotope ratio mass spectrometer (IRMS, Thermo Scientific, Waltham, MA, USA). The methanol used during PLFA conversion to FAMEs was isotopically characterized and δ^13^C values for PLFAs were corrected via the relationship

δ^13^C_PLFA_ = [(N + 1) × δ^13^C_measured_ − δ^13^C_MeOH_]/N (1)

where N is the number of carbon atoms. Individual samples were analyzed in triplicate and precision is reported as the mean ± one s.d. All δ^13^C values are reported in standard delta notation relative to PDB.

Bulk organic material and carbonate samples were collected from the microbial surfaces of three separate structures and each depth and freeze-dried. Fourteen meters was the exception, where three samples were taken from the same microbialite. Carbonate stable isotope analyses were performed at McMaster University using an Optima isotope ratio mass spectrometer with an Isocarb acid bath at 90 °C. Bulk organic samples were treated with HCl to remove any residual carbonate and δ^13^C_org_ was determined using an EA-Delta XL at McMaster University. All δ^13^C values are reported in standard delta notation relative to PDB ± one s.d.

#### 2.3.4. Scanning Electron Microscopy and Confocal Laser Scanning Microscopy

Samples were defrosted overnight in a 2% gluteraldehyde solution and prepared for imaging using the procedure described by Ryter and Kellenberger [43] and Omelon et al. [44]. Specifically, samples were washed with 0.1 M sodium cacodylate buffer (pH 7.3), fixed with 0.1% osmium tetroxide/0.1 M sodium cacodylate buffer, washed with 0.1 M sodium cacodylate buffer, dehydrated through a graded ethanol (EtOH) series (25%, 50%, 75%, and 100% EtOH), and embedded in LR White acryclic resin. These were cut, mounted, and polished into thin sections. Confocal laser scanning microscopy was performed using a Zeiss LSM 5 Duo at the Biotron, The University of Western Ontario. Reflected light images were taken with the 488 nm laser and Long Pass 420 emission filter. Fluorescent images were taken with the 543 nm laser and Long Pass 560 emission filter to capture the fluorescence of antenna pigments associated with cyanobacteria in the reaction centers of Photosystem II [45,46,47]. Samples were coated with osmium prior to visualization with the LEO 1540XB field emission gun scanning electron microscope (SEM) equipped with secondary electron, in-lens, and quadrant back scattering detectors.

#### 2.3.5. DNA Extraction, Purity and Concentration Measurements

The microbialites used in the PLFA analysis were also used for DNA extraction. DNA was extracted on-site using the MoBio Powersoil DNA extraction kit (Mobio, Carlsbad, CA, USA), using ~10 g of homogenized integrated microbialite. DNA concentrations were determined on-site using a Nanodrop-3300 (ThermoFisher, Nandrop Wilmington, DE, USA) with PicoGreen^®^ reagent according to the manufacturer’s instructions (Invitrogen, Carlsbad, CA, USA). DNA purity was determined by absorbance (260/280 and 260/230 ratios) using a Nanodrop-1000 (ThermoFisher, Nandrop Wilmington, DE).

#### 2.3.6. Quantitative PCR Measurements and Standard Curve Construction

Total bacterial 16S rDNA gene copy numbers were determined using the standard curve method with the primers Eub338f/518r as outlined in Fierer et al. [48] and Waldrop et al. [49]. Total cyanobacterial 16S rDNA copy numbers were also determined using the standard curve method [48] and custom cyanobacterial primers designed for this study; specifically, Cya106f and a modified Cya359r primer [50] (Table 1). Primer-BLAST was used to confirm Cya106f/359r targets as predominantly cyanobacteria. Both total bacterial and total cyanobacterial 16S rDNA copy numbers were normalized to the dry weight of extracted microbialite. All qPCR experiments were performed on an IQ5 instrument (BioRad, Hercules, CA, USA) using Evagreen sso7d master mix (BioRad, Hercules, CA) and a 0.3 µm final concentration of primer. Thermocycling parameters were modified to use the faster Evagreen sso7d master mix for both Eub338f/518r and Cya106f/359r primer sets. The thermal profile for the both primer sets was 98 °C (2 min; initial denaturation-1 cycle), followed by 50 cycles at 98 °C (5 s), with one annealing/extension step of 53 °C (30 s) for Eub338f/518r and 60 °C (30 s) for Cya106f/359r, with subsequent melt curve analysis from 50–98 °C at 0.5 s intervals. Standard curves were created by amplifying the 16S rDNA of interest (either Cya or Eub) and purification of the product by Mini-Elute Column (Qiagen, Germantown, MD, USA), then were further examined by agarose gel analysis for correct size. Standard purities were determined by Nanodrop-1000 absorbance ratios and concentrations by Nanodrop-3300 using Picogreen^®^ reagent. Strict adherence to the MIQE guidelines was followed during this study [51].

#### 2.3.7. Statistical Analyses

The weight % of total organic carbon (TOC), mean PLFA concentrations, total bacterial 16S copy numbers, and cyanobacterial 16S copy numbers were all compared over sample depths by one-way ANOVA, and significant differences were determined using Tukey’s HSD post hoc test in IBM SPSS 20 (IBM Corp., Armonk, NY, USA). In some cases, the data were log transformed prior to statistical analysis to account for unequal variance in the means. The Shapiro–Wilk test was used to check that the data were normally distributed.

## 3. Results

### 3.1. Physical Properties of Kelly Lake

**Figure 2:**
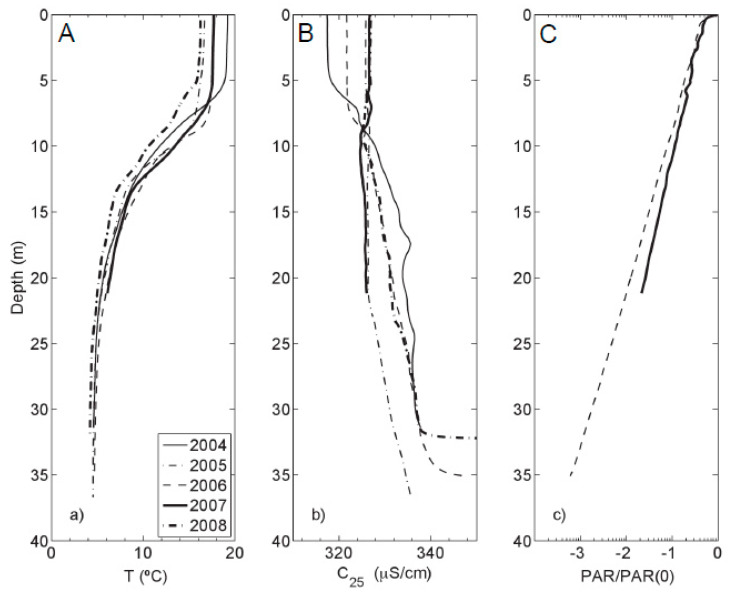
(**a**) Temperature; (**b**) Specific conductance; and (**c**) photosynthetically active radiation (PAR) profiles (logarithmic scale) of Kelly Lake with depth. All profiles were measured during the first week of August, except 2008, which was measured in the first week of July. Only PAR profiles for 2006 and 2007 are plotted in panel (**c**).

Underlying a surface-mixing layer, the reported seasonal thermocline was located each year between 5 and 15 m [37], which is consistent with Ferris et al. [20]. Below the thermocline is a bottom layer with mild thermal stratification (Figure 2a).

Specific conductance profiles (Figure 2b) generally displayed an increase in conductivity with depth. In 2005, specific conductance varied little between the surface waters and 20 m, but still showed an increase in the near-bottom waters. PAR profiles (Figure 2c) decreased exponentially with depth. The one percent light level was between 22 and 25 m of depth.

### 3.2. Isotopic Composition of DIC and Microbialite Carbonate

Using a δ^13^C value of atmospheric CO_2_ of −9.6 ± 0.2 ‰ and a temperature range of 0 to 20 °C, Brady et al. [52] calculated that DIC in isotopic equilibrium with the atmosphere near Kelly Lake should have a measured δ^13^C value between 1.1‰ and −1.3‰. The summer surface water δ^13^C_DIC_ values of Kelly Lake were ^13^C-depleted with respect to this range (mean δ^13^C_DIC_ −4.9 ± 1.1‰) and indicate that the DIC was not in isotopic equilibrium with the atmosphere. This isotopic depletion implies that there are considerable inputs of ^13^C-depleted inorganic carbon from in situ respiration, groundwater and/or surface water. Samples taken in 2006 from groundwater wells near the northern and southern tips of the lake, “Jim well” and “Guy and Ursula well”, respectively (Figure 1), both had δ^13^C_DIC_ values of −10.1‰, indicating that the local groundwater δ^13^C_DIC_ is depleted by −5.2‰ relative to the mean δ^13^C_DIC_ of Kelly Lake. The surface water inflow from Porcupine Creek, collected in 2006, had a δ^13^C_DIC_ value of −7.3‰ (Table 2). Both sources could account for the observed δ^13^C_DIC_ disequilibrium. From the depth profile taken in 2012, the δ^13^C_DIC_ values tend to decrease with depth. The δ^13^C_DIC_ at 15 m and 30 m were found to be −6.7‰ and −7.2‰, respectively (Table 2); values which were 1.0‰ and 1.5‰ more negative than the δ^13^C value for surface water DIC measured that year. Previous attempts at measuring dissolved organic carbon (DOC) within Kelly Lake found that concentrations were often below detection (<0.5 mg/L) [37].

The Δ^14^C value of Kelly Lake surface water DIC was found to be −107‰. This was converted to an age estimate of 1190 ± 30 years (cal BC/AD) using OxCal version 4.1 (IntCal09) [40].

The δ^13^C_carb_ values of microbialite carbonates were consistent at a given depth and were very similar between depths of 11, 14, and 20 m, with values of −2.7‰ ± 0.5‰, −3.8‰ ± 0.1‰, and −3.8‰ ± 0.5‰, respectively. Carbonates from 26 m were less ^13^C−depleted, with an average δ^13^C_carb_ of −0.3‰ ± 0.5‰ (Table 3).

### 3.3. Isotopic Composition of Microbialite Bulk Organic Material

The δ^13^C values of bulk organic carbon from microbialites at all depths (11 to 26 m, Table 3) ranged from −27‰ to −34 ‰ with an average δ^13^C_org_ of −30.6‰ ± 2.7‰ (*n* = 12). This corresponds to an average offset of −25.7‰ between the δ^13^C values of the organic material and the average surface water DIC and is consistent with non-CO_2_-limited C_3_ photosynthesis [53]. The 26 m samples contained the most ^13^C-depleted organics with an average δ^13^C_org–DIC_ offset of −29.3. If the DIC at 30 m was consistently depleted by −1.5‰ from surface waters, as measured in the 2012 depth profile, the δ^13^C_org–DIC_ offset would be closer to −27.8‰.

### 3.4. PLFA Concentrations and % Total Organic Carbon

**Figure 3:**
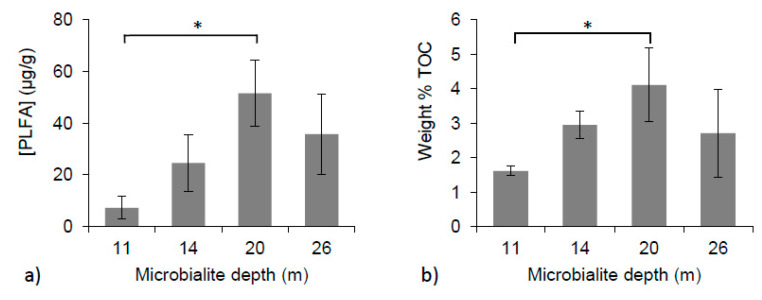
(**a**) Concentrations of PLFAs over microbialite depths (one-way ANOVA, *p* < 0.01); (**b**) Weight % TOC of microbialite surface material with depth (one-way ANOVA, *p* < 0.05). * Indicates significant differences between depths.

PLFA concentrations varied between individual microbialite structures and with depth. Microbialites collected from 11 m had an average concentration of 7.2 ± 4.4 micrograms of PLFAs per gram of dry sample extracted (µg PLFA/g), and samples from 14 m had a mean of 24.5 ± 11.0 µg PLFA/g. The highest concentrations of PLFA, 51.5 ± 12.7 and 35.7 ± 15.6 µg PLFA/g, were extracted from samples growing at 20 and 26 m, respectively (Figure 3a). A significant difference in PLFA concentrations was found between 11 and 20 m (one-way ANOVA, *p* < 0.01).

Using the conversion factor of 2.6 × 10^4^ cells/pmol PLFA [54] and the mass of material extracted, the total concentrations of PLFA can be converted into estimates of cell densities. In general, cells densities ranged from 2.3 × 10^8^ to 5.9 × 10^9^ cells/g and were found to be the highest at 20 m, with a mean of 4.8 × 10^9^ ± 1.2 × 10^9^ cells/g.

Within the microbialite surface material, the weight % of TOC was also found to have a significant difference amongst the means (one-way ANOVA, *p* < 0.05). The lowest mean weight % TOC value was found at 11 m (1.6% ± 0.1 %) and the highest at 20 m (4.1% ± 1.1 %). Samples collected from 14 and 26 m had mean weight % TOC vales of 3.0% ± 0.4 % and 2.7% ± 1.3 %, respectively (Figure 3b).

### 3.5. PLFA Profiles

**Figure 4:**
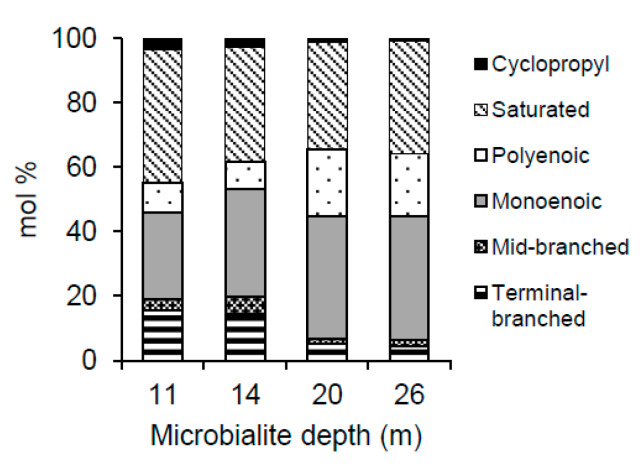
The mol % of each structurally defined PLFA class as a function of depth.

For all samples, the saturated, straight-chained PLFAs and the monoenoic PLFAs were the most frequently found in the PLFA profiles (see Figure 4 and Table 4 for a representative sample of each depth). Saturated straight-chained PLFAs were found to range in size from 14:0 to 24:0, however, the longer chained species characteristic of eukaryotic organisms, that is >20:0 [55], were only found in the deeper microbialite samples (20 and 26 m). The dominant saturated PLFA in all samples was 16:0, comprising from 17.1 to 29.7 mol % of the total PLFAs. Over all depths, the sum of all monoenoic PLFAs ranged from approximately 20 to 45 mol % and consisted largely of 16:1 (mostly 16:1Δ9) and 18:1 (mostly 18:1Δ9), lipids which are generally associated with gram-negative bacteria such as cyanobacteria [56,57]. Branched PLFAs were consistently more abundant in the 11 and 14 m samples, with 10me16:0 as well as *i-*15:0, and *a*-15:0 being the dominant components of this PLFA type. Although polyenoic PLFAs were present in all microbialites, they were only found in proportions greater than 15 mol % in the 20 and 26 m samples. Cyclopropyl 17:0 and 19:0 displayed minor decreases in mol % with depth, although these PLFAs totaled less than 3.5 mol % at all depths and therefore represented minor components of the extracted PLFAs (Figure 4).

### 3.6. Isotopic Composition of Microbialite PLFAs

The isotopic compositions of the major microbialite PLFAs were determined for those that could be baseline resolved on the GC-IRMS system (see Table 4 for a representative sample from each depth). At all depths, *i-*15:0 and *a-*15:0 had a higher δ^13^C value than all the other PLFAs which were analyzed, falling within the δ^13^C range of −30‰ to −40‰. 14:0 and 16:0, as well as all the unsaturated PLFAs, were highly depleted, with δ^13^C values ranging from −37‰ to −50‰. Some variations in the δ^13^C_PLFA_ values were found between the triplicate samples from each depth, although most differences were less than 2‰.

**Figure 5:**
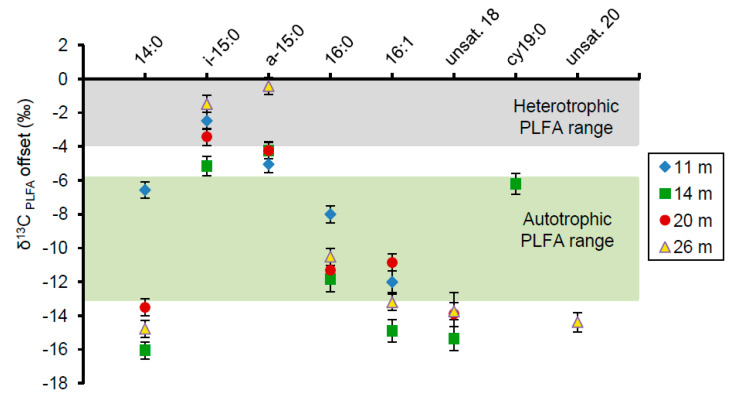
The δ^13^C offset of PLFAs from one representative sample at each depth relative to the δ^13^C value of the bulk organic carbon, set here as 0. Error bars represent one standard deviation from the mean based on triplicate analysis of the same sample. Most PLFA, except for i-15:0 and a-15:0, fall within or below the photoautotrophic synthesis range.

Relative to the average bulk organic carbon δ^13^C value determined at each depth (Table 3), the major PLFAs were depleted in ^13^C (Figure 5). The saturated, monoenoic, and polyenoic PLFAs at all depths were highly depleted relative to the bulk organic carbon by values from 8‰ to 16‰. These depletions are generally characteristic of synthesis by autotrophs such as cyanobacteria [34,58]. In contrast, the branched PLFAs *i-*15:0 and *a-*15:0 were fractionated by approximately 0‰ to 6‰, consistent with aerobic heterotrophic synthesis [31,33,59,60].

### 3.7. Microbial Community Relative Abundance

**Figure 6:**
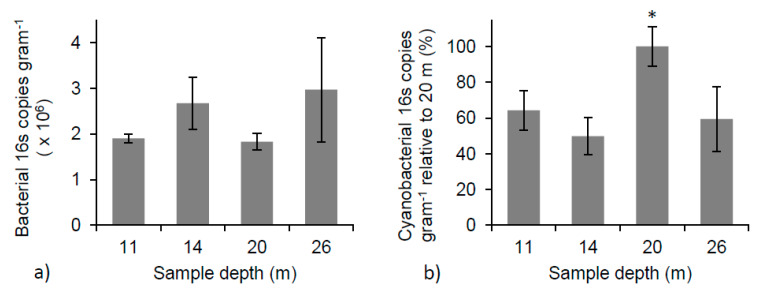
(**a**) Total bacterial 16S copy number per gram (dry weight) of microbialite shows no significant change with depth (one-way ANOVA); (**b**) The percent of cyanobacterial 16S copy number at each depth relative to 20 m. * Indicates a significant difference between 20 m and all other depths (one-way ANOVA, *p* < 0.05 for all).

The relative abundance of bacterial 16S copies varied from 1.9 × 10^6^ ± 9.9 × 10^4^ 16S copies gram^−1^ of microbialite (dry weight) to 3.0 × 10^6^ ± 1.1 × 10^6^ 16S copies gram^−1^, and displayed no significant change with depth (Figure 6a). The variation in bacterial abundance at each depth illustrates the patchy and heterogeneous nature of the microbial community across the surface and into the microbialite structures. The relative abundance of cyanobacterial 16S copies gram^−1^ showed an increase as a function of depth, reaching a maximum at 20 m. At the 20 m depth, the average number of cyanobacterial 16S copies gram^−1^ was significantly different than those found at 11, 14, and 26 m (one-way ANOVA, *p* < 0.05 for all). The mean number of cyanobacterial 16S copies gram^−1^ at 20 m was double that at any other depth (Figure 6b), suggesting a change in cyanobacterial species or abundance is occurring at this depth.

### 3.8. Microscopy

**Figure 7:**
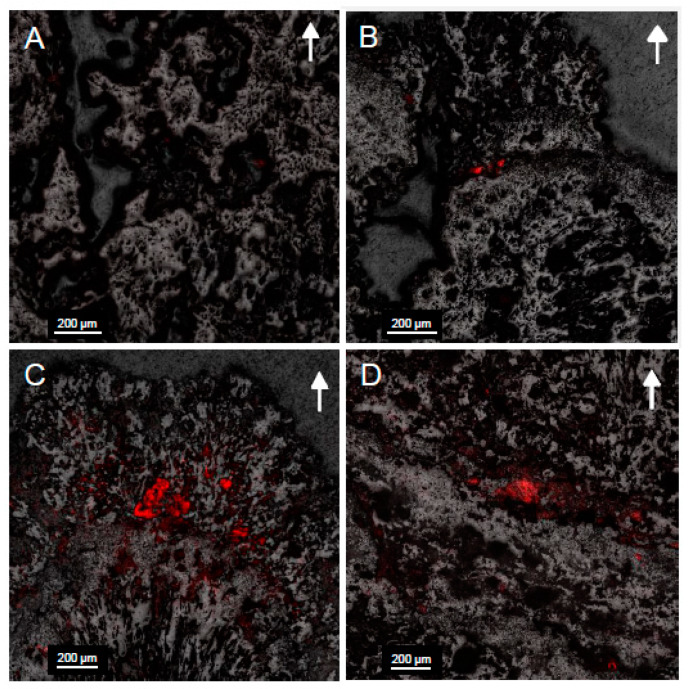
Confocal images of microbialite surfaces in cross section from samples collected at (**A**) 11 m; (**B**) 14 m; (**C**) 20 m; (**D**) 26 m. The fluorescent overlay indicates the presence of cyanobacteria within voids located in the first few millimeters of carbonate minerals. Arrows indicate the orientation of microbialite growth. Note the greater fluorescence and therefore higher abundance of cyanobacteria at 20 m.

**Figure 8:**
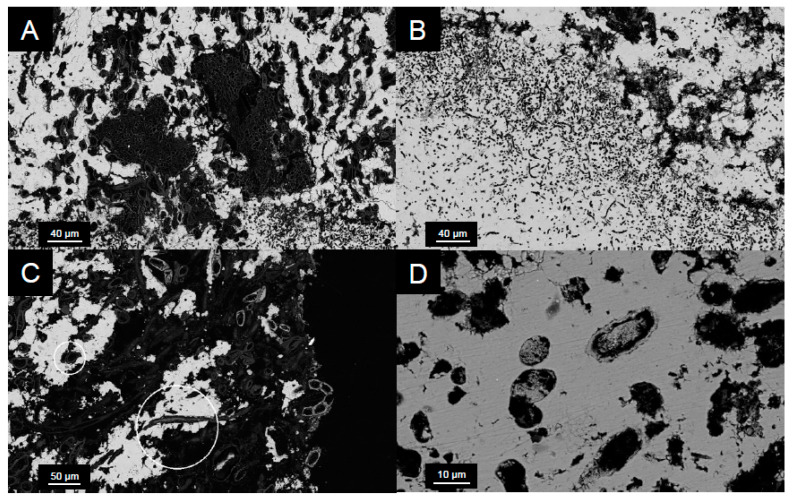
SEM back-scattered electron micrographs of microbialites from 20 m in cross section. (**A**) Coccoid cyanobacterial microcolony located less than 1 mm below the microbialite surface. (**B**) Void spaces within the carbonate are similar in shape and size to extant coccoid and filamentous species. (**C**) Cells encased in carbonate minerals (circled in white) suggest that in situ precipitation occurred around individual cells. (**D**) Secondary infilling of void spaces with carbonate minerals suggests active precipitation.

Confocal images (Figure 7) displayed an increase in fluorescence within microbialite samples collected from deeper in Kelly Lake. This was particularly the case at 20 m, where the greatest fluorescence, and thus the highest abundance of cyanobacteria, were found. Fluorescence was only found in the upper 5 mm of microbialite carbonate, which displayed cells encased in carbonate (Figure 8).

## 4. Discussion

### 4.1. Isotopic Biosignatures in Microbialite Carbonate

**Figure 9:**
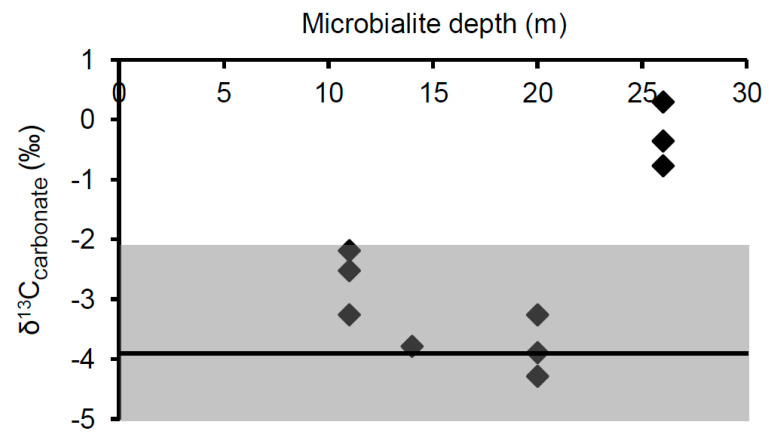
δ^13^C values of surface carbonates collected from microbialites found at depths of 11, 14, 20, and 26 m. The shaded area represents the predicted range of δ^13^C_carb_ values which would result from equilibrium precipitation, based on the most enriched and depleted surface δ^13^C_DIC_ values found in any of the summer sampling years. The solid line represents the mean predicted δ^13^C_carbonate_ value determined from surface water δ^13^C_DIC_ values.

Since calcite becomes enriched in ^13^C by 1.0‰ ± 0.2‰ when precipitating abiotically from bicarbonate [61], the average surface water δ^13^C_DIC_ of −4.9‰ ± 1.1‰ for Kelly Lake predicts an equilibrium δ^13^C_carb_ value of −3.9‰ ± 1.3‰. The maximum range of δ^13^C_carb_ values predicted for equilibrium precipitation (−2.2‰ to −5.3‰) was determined based on the most enriched (−3.2‰, measured June 2008) and depleted (−6.3‰, measured June 2006) δ^13^C_DIC_ values found in any of the summer sampling years. The measured δ^13^C_carb_ values of microbialites from 11 to 20 m, displayed in Figure 9, fell within the predicted equilibrium range. This implies that in these samples, microbial activity is not significantly influencing the local DIC isotopic composition, and thus no isotopic biosignatures were found.

In contrast, carbonates from all samples collected at 26 m were ^13^C-enriched relative to the mean summer δ^13^C_DIC_, with δ^13^C_carb_ values ranging from 0.3‰ to −0.8‰. This enrichment is likely an isotopic biosignature which reflects photoautotrophic influences within the surface biomass during precipitation of the microbialite carbonate. In relation to the mean value predicted for abiotic precipitation from surface waters, the average δ^13^C_carb_ value from 26 m is enriched by 3.6‰. Similar changes in isotopic composition due to photosynthetic activity have been reported from cultivation studies [19] and from measured differences in δ^13^C_carb_ value between material precipitated in association with active cyanobacterial layers (potentially containing microalgals) versus heterotrophic layers of artificial mats [23]. δ^13^C_carb_ values that are ^13^C-enriched above equilibrium by ~2 to 3.5‰ is also consistent with biosignatures identified in Pavilion Lake microbialites [8,24,35]. If the trend seen in the 2012 depth profile of more depleted δ^13^C_DIC_ values at depth was also present when these samples were collected, this isotopic enrichment would be even larger (~5.1‰). Similar to the isotopic signatures found in Pavilion Lake by Brady et al. [8], the δ^13^C enrichments found at 26 m are not thought to result from lake-wide shifts in δ^13^C such as those observed during whiting events [62]. Water clarity has not only remained unchanged, but the lack of such enrichments at all other depths precludes the possibility of a lake-wide event.

The observation of this isotopic biosignature only in the deepest sample was unexpected. While previous results have shown that such enrichments are predominantly associated with cyanobacteria-dominated surface nodules [8] and structures at shallow (<11 m) depths [35,36], there have been few observations of such a biosignature for microbialite surface carbonates at a greater depth in Pavilion Lake, although some have been noted [24]. Further, the lack of isotopic enrichment in the shallower depths in Kelly Lake made this observation all the more unexpected. It is worth noting that an isotopic biosignature was not found at 20 m, where the highest concentrations of PLFAs, cyanobacterial-specific 16S copies, and presumably the highest biological activity, were observed. This may be due to differences in the balance of photoautotrophic and heterotrophic influences on the isotopic composition of the local DIC at this depth; a balance that has been suggested to impact the creation and preservation of isotopic biosignatures in Pavilion Lake [24,35]. In particular, Belan and colleagues hypothesized that a shift in the balance of photoautotrophic vs. heterotrophic metabolisms was responsible for the loss of observable ^13^C-enriched biosignatures when transitioning from the exterior surface of microbialites to the interior [24]. Alternatively, more strongly isolated microenvironments may occur at 26 m, which allows isotopic enrichment of the DIC pool from which the carbonate is being precipitated. Regardless, the lack of an isotopic signature at depths above 26 m does not imply that photoautotrophic processes are necessarily irrelevant to microbialite building in these locations. Rather, it indicates that a signature of this process is not being preserved.

Interestingly, small cm-scale microbialites (thrombolites) previously collected by Ferris et al. [20] from less than 1 m below the Kelly Lake surface were found to have δ^13^C_carb_ values enriched above equilibrium. At these shallow depths, ^13^C-enrichments of 4.6‰ to 5.2‰ above the corresponding DIC were found [20], values consistent with those discovered at 26 m in the current study, while both stromatolites and thrombolite structures are found within Kelly Lake [20]. These softer stromatolites were more similar to the small, surface nodules studied by Brady et al. [8] than to the thin biofilm covering the larger structures examined in this study. As such, the biosignatures observed in the stromatolites by Ferris et al. [20] may be the result of a more isolated microenvironment within such structures, as pointed out by Brady et al. [8], while the effects on the local environment may not be sufficient to create a biosignature in the harder microbialites growing at depth studied here.

### 4.2. Predominance of Photoautotrophic Microorganisms

The PLFA profiles and the isotopic composition of the microbialite PLFAs indicate that both photoautotrophic and heterotrophic microorganisms are actively growing on or within the first couple millimeters of the microbialite surfaces. In general, the PLFA profiles suggest that photoautotrophic microorganisms are predominant, yet heterotrophs are present and may still play a role in altering the local geochemistry and/or influencing carbonate precipitation. The straight-chained saturated species such as 14:0, 16:0, and 18:0 are ubiquitous across most bacteria and therefore cannot be applied as direct biomarkers for particular bacterial groups [63,64]. Monoenoic PLFAs are attributed to the presence of cyanobacteria [65,66], although other gram-negative species such as members of the genus *Rhodomicrobium* have also been found to synthesize high concentrations of these PLFAs [41,67]. The high abundance of monoenoics, including 16:1 and 18:1, are consistent with contributions from cyanobacteria at all depths. Polyenoic PLFAs such as 16:2, 16:3, 20:4, 20:5, 22:5 all become more abundant below 14 m and are associated with cyanobacteria or phototrophic microeukaryotes such as algae and diatoms [68,69,70,71], whereas 18:2 is attributed to fungi [72]. While cyanobacterial and microbial abundances altered with depth, due to the lack of 16S rRNA we cannot suggest community structural change with depth.

Biomarkers of heterotrophic microbes such as 10me16:0, *i*-15:0, and *a*-15:0 were found in all samples, but generally at low relative proportions. *i*-15:0 and *a*-15:0 are PLFAs characteristic of gram-positive and gram-negative bacteria, but are not typically found in cyanobacteria [57,73,74]. Their presence indicates the potential influence of heterotrophic microbes on the local geochemistry and thus on carbonate precipitation. Heterotrophic influences on carbon formation have been noted in other systems [1]. In Kelly Lake, heterotrophic contribution to precipitation is speculative and biosignatures of heterotrophic influence on carbonate precipitation were not identified, indicating that if there are heterotrophic contributions, they are not detectable. With abundances consistently higher at 11 and 14 m, these branched PLFAs suggest the relative proportion of the heterotrophic community in Kelly Lake decreases with depth. 10me16:0, a biomarker for sulphate-reducing bacteria [66,75], shows the same trend. Cyclopropyl PLFAs such as cy17:0 and cy19:0 typically come from anaerobic heterotrophs and were only found in minor concentrations. These cyclopropyl fatty acids have, however, also been found in cyanobacteria [72,76]. *Rhodomicrobium* are active photoheterotrophic organisms which can, in theory, alter the saturation index of calcium carbonate through anoxygenic photosynthesis [13]. A photoheterotroph (Candidatus *Chloracidobacterium thermophilum*) has been identified in Pavilion Lake microbialites [36], however the role, if any, of this organism in carbonate precipitation is unknown. If they are contributing some of the observed monoenoic PLFAs, then the presence of heterotrophs may be underestimated by looking solely at the PLFA profiles.

The isotopic offsets of PLFAs relative to the bulk organic carbon were consistent with a predominance of photoautotrophic synthesis at all depths, with some evidence of heterotrophic activity (Figure 5). This included the abundant saturated straight-chained PLFAs 14:0 and 16:0, which displayed offsets as large as −16‰. Although these particular fatty acids are not normally attributed to specific bacteria, such large offsets link them to a photosynthetic origin and therefore support the predominance of autotrophy in the overall microbial community. These larger offsets are also consistent with values observed in Pavilion Lake and attributed to photoautotrophic synthesis [35]. Monoenoic PLFAs displayed similar isotopic depletions, ranging from −7.5‰ to −13.5‰, which correlate with offsets found in cyanobacteria [34,58]. The δ^13^C_PLFA-biomass_ offset of cy19:0 at 14 m ranged from −6.2‰ to −9.8‰ and could be attributed either to cyanobacterial synthesis or anaerobic heterotrophy [34,60,72]. *i-*15:0 and *a-*15:0 were the only PLFAs analyzed to fall within the aerobic heterotrophic fractionation range of roughly 0–4‰, although some of these measurements were slightly more depleted [33,59,60].

The abundance of cyanobacteria associated with the surfaces of the microbialites, particularly at 20 m, included both coccoid and filamentous species (Figure 7 and Figure 8). This finding is consistent with previous observations from shallow (<1 m) microbialites in Kelly Lake [20] and with observations from nearby Pavilion Lake [77,78]. Those which are coccoid in nature were deeply encrusted within the first 0–3 mm, forming the base of a thin biofilm that covers the microbialite and are morphologically similar to *Gloeobacter*, *Gloeocapsa*, *Synechoccocus* or *Cyanothece*. In addition to having been visually identified in Kelly Lake thrombolites [20], *Synechoccocus* are associated with whiting events and ^13^C-enrichments in carbonates [62] and are the best candidates for these deeply encrusted coccoid cells. Very thin filaments, either ball-chain type or non-ball chained type, were also observed using confocal microscopy on the 20 m microbialites. The ball-chain type cyanobacterial filaments morphologically resemble members from the order Nostocales, including the genera *Scytonema, Rivularia* or *Nostoc*. The non-ball-chain type cyanobacteria morphologically resemble members from order Oscillatoriales, including the genera *Schizothrix*, *Planktothrix*, *Leptolyngbya*, and *Phormidium*. The observation of these cyanobacterial species confirms that photoautotrophic organisms are present in abundance and potentially contributing to active microbialite growth. Members of these genera have been identified previously in Pavilion Lake [9,36,77].

### 4.3. PLFA and Biomass Distribution

PLFA concentrations on Kelly Lake microbialite surfaces were anticipated to decrease with increasing sample depth because of declining light intensity (Figure 2c), and thus less favorable growth conditions for photosynthetic organisms. However, the highest concentrations of PLFAs were found associated with the deeper samples analyzed (20 and 26 m). Tukey’s HSD post hoc test determined that PLFA concentrations and weight % TOC were significantly different between the depths of 11 and 20 m (one-way ANOVA, *p* < 0.01 and *p* < 0.05, respectively). Cell density estimates (max. 5.9 × 10^9^ cells/g at 20 m) apply only to the outer 2–3 mm of microbialite that was sampled during PLFA analysis, as this is where the vast majority of biomass exists. It is worth noting that there is error associated with the use of a generic conversion factor [28,79], particularly if there is variation in cell sizes with depth. Notwithstanding these limitations, the observation of increased PLFA concentrations at depth were also concurrent with increases in TOC concentrations in the deeper samples (Figure 3b), and observations of cyanobacterial cells by confocal microscopy (Figure 7) also showed evidence of higher cell density at deeper depths.

In contrast to the PLFA results, the average abundance of general bacterial 16S copies gram^−1^ was not significantly different between any depths. Assuming one copy of 16S rDNA per cell, qPCR estimates of cell abundances are 2–3 orders of magnitude lower than those determined using PLFA. This large difference in cell density estimates may be a result of differences in measurement techniques and/or biases that are a result of inherent limitations of the qPCR technique [80,81,82]. The comparison between qPCR- and PLFA-based results in Kelly Lake is also likely affected by the fact that 16S qPCR measurements only provide estimates for total bacterial abundance, whereas PLFA estimates include all bacterial and eukaryotic cells. Furthermore, despite efforts to only sample the outer microbialite material, sampling effects could also have contributed to some of the observed difference because the internal microbialite material is generally less populated by microbes [78].

The relative abundance of cyanobacterial 16S copies gram^−1^ reveals a shift in the cyanobacterial community at depth, particularly at 20 m, where a significantly higher number of cyanobacterial 16S copies gram^−1^ was found compared to all other depths (one-way ANOVA, *p* < 0.05 for all) (Figure 6b). This shift was also observed in confocal images (Figure 7), displaying a higher abundance of cyanobacteria at 20 m relative to all other depths. Furthermore, notable changes in the PLFA profiles were seen between 14 and 20 m, with a decrease in branched species and an increase in polyunsaturated PLFA (Figure 4), indicating that a shift in the microbial community occurred. This increase in cyanobacterial-specific biomass at 20 m could explain the PLFA- and TOC-based observation of increased total biomass at depth.

The observed increase in biomass at depth may be a result of more favorable growth conditions at 20 m as compared to 11 m. Phosphorous, a biologically-essential nutrient, which is known to inhibit microbial growth at low concentrations [83], provides a potential explanation for this phenomena. Although Kelly Lake is ultraoligotrophic at all depths, an increase in phosphorous concentrations has been observed with depth, specifically an increase from 2 to 4 µg/L between the surface water and 20 m (PLRP 2005 measurements, unpublished data). If such a gradient in phosphorous concentrations existed in the year the microbialites were sampled, it may have been sufficient to result in the observed increases in PLFA concentrations and microbial biomass. In other phosphorous-limited aquatic systems, increasing phosphorous concentrations by 2-5 times has been shown to significantly amplify total microbial biomass (by ~2 times), growth efficiency, and bacterial activity, particularly if the system is oligotrophic [84,85,86]. Alternatively, the increase in biomass at 20 m may be a result of more favorable light conditions for low-PAR cyanobacteria, or due to unconstrained factors such as sedimentation rates, levels of eukaryotic grazing, or effects of the thermocline.

The decrease in δ^13^C_DIC_ in Kelly Lake deepwater, as observed in the 2012 depth profile, indicates the occurrence of either increased respiration or increased inputs of local groundwater at depth, which could result in higher phosphorous concentrations. “Jim well” and “Guy and Ursula well” were found to contain phosphorous concentrations roughly three times higher than the lake surface waters and may be the source for additional phosphorous at depth [37]. The increase in specific conductance with depth in Kelly Lake (Figure 1) provides additional support for groundwater input, since the two nearby wells were found to be 1.3 to 2 times more saline than Kelly Lake surface waters. Groundwater inputs are likely altering the chemical properties of the deepwater relative to the surface and may be the root cause of the higher biomass observed at depth, but without constraint on the hydrologic regime it is impossible to eliminate contributions from increased microbial respiration at depth, or the other unconstrained factors mentioned above, as potential components of the explanation.

### 4.4. Comparison to Pavilion Lake

The phospholipid fatty acid profiles, carbon isotopic signatures, and carbon sources have all been previously investigated for Pavilion Lake [8,35,52,87]. Lim et al. [37] have also provided a detailed description of the limnology for both Pavilion and Kelly Lakes. It appears that in both systems, autotrophy is the metabolism that dominates the microbialite-associated microbial communities [8,35]. The PLFA profiles were quite similar in that they were primarily composed of straight-chained, saturated PLFAs and monoenoic PLFAs of the same type [8,35,52,87]. In addition, most of the PLFAs were highly fractionated relative to the bulk organic carbon δ^13^C values, and thus appeared to be synthesized by photoautotrophs [8,35]. However, key differences were found between these two systems, which emphasize their uniqueness, including their distribution of PLFA concentrations with sample depth, the δ^13^C_DIC_ and Δ^14^C_DIC_ values, and the observation of an isotopic biosignature at 26 m in Kelly Lake samples.

Unlike Kelly Lake, an increase in PLFA concentrations with depth has not been observed during the summer months in Pavilion Lake. On the contrary, Brady [35] found that during summer months, shallower microbialites consistently had higher PLFA concentrations and thus greater cell densities. It is possible that in Pavilion, the influences of local groundwater, surface water, and/or respiration inputs on the summertime lake chemistry are not as significant, and/or do not influence microbial growth at depth to the same extent as in Kelly Lake. The fact that the δ^13^C_DIC_ value of surface water in Pavilion was almost always in isotopic equilibrium with the atmosphere [8], whereas that of Kelly consistently fell outside of the isotopic equilibrium range, exemplifies the greater influence of groundwater, surface water, and/or in situ respiration inputs on Kelly Lake surface water chemistry compared to Pavilion. This is also supported by the older age of the surface water DIC in Kelly Lake (~820 years) relative to Pavilion Lake (~440 years; [52]), which suggests that surface/groundwater inflows are strongly influencing the isotopic composition of the DIC in Kelly Lake. In Pavilion Lake, inputs of local groundwater, with similar δ^13^C_DIC_ values to the Kelly Lake wells, in conjunction with in situ respiration of organic material, were observed to have no effect on the δ^13^C of the bulk lake surface water [52]. This suggests that, all other factors being equal, significant inputs of surface water from Porcupine Creek are likely the primary driver of the observed isotopic depletion of Kelly Lake surface water DIC.

Groundwater and/or in situ respiration are likely controlling changes in the lake chemistry at depth in Kelly Lake, such as phosphorous concentrations, since inputs from Porcupine Creek to the deepwater are improbable during the summer because the inflow is unlikely to penetrate the seasonal thermocline that develops. Groundwater and/or in situ respiration were also determined to influence the deepwater in Pavilion [52], however, phosphorous concentrations in Pavilion Lake have not shown the consistent trend of increasing with depth in the central basin, where the microbialites were collected, despite variations in these levels between seasons [37]. The exception was at 59.5 m in the deepwater, where phosphorous concentrations were generally found to be greater than surface values [37], but groundwater input was also estimated to be slightly greater [52] and microbial growth was likely limited by other factors, such as light availability.

The isotopic enrichments found in the carbonate from 26 m samples in Kelly Lake were not largely confined to nodule structures on the microbialite surfaces like those present at some depths in Pavilion Lake. Instead, δ^13^C values were measured for the bulk surface carbonate because distinct nodules like these were not present on the sampled structures. As reported by Brady et al. [8], isotopic biosignatures were observed in almost all of the nodule samples analyzed from 10 to 21 m of depth. Similar signatures were observed in bulk surface carbonates from Pavilion Lake, predominantly at 11 m, but were also observed in some samples from 20 and 26 m [24,35]. Such a signature was lacking in all of the shallow (≤20 m) samples analyzed from Kelly Lake. Within Pavilion Lake nodules and surface carbonates, photoautotrophic influences were either greater at these depths relative to Kelly or the nodular/biofilm microenvironment was more conducive to alteration of the local geochemistry. The fact that broadly occurring biosignatures in the bulk microbialite carbonate were observed at shallow depths in Pavilion and 26 m in Kelly suggests specific growth conditions, such as PAR levels or phosphorous concentrations, may have implications for the production of these isotopic biosignatures, and therefore may have been factors influencing their creation and preservation in ancient microbialites.

## 5. Conclusions

PLFA profiles, Δδ^13^C_PLFA-org_ offsets, cyanobacterial 16S copy numbers, and isotopic measurements of the bulk surface carbonates in Kelly Lake identify autotrophy as the predominant metabolism of the microbialite-associated microbial communities. The PLFA profiles were dominated by straight-chained, saturated PLFAs and monoenoic PLFAs, which were determined to come from a photoautotrophic source based on their high levels of isotopic depletion. Carbonates enriched in ^13^C outside of the predicted abiotic precipitation range were found in microbialites from 26 m and represent a biosignature for photoautotrophic influence on the local geochemistry at this depth. This enrichment was not observed in any of the carbonates collected from shallower depths. Whether photoautotrophic metabolisms influence carbonate precipitation throughout the entire lake is not evident through these analyses, however, it is likely that photoautotrophic influences dominate at the depths where no isotopic signature was found, but are simply not significant enough to result in a discernible isotopic biosignature.

An interesting feature of the Kelly Lake microbialites was the tendency of their PLFA concentrations to increase with depth, with a significant difference between 11 and 20 m. This contrasts Pavilion Lake microbialites, which generally had decreasing concentrations of PLFAs with depth, presumably due to the decreasing light intensity and thus lower cellular abundances. In Kelly Lake, this phenomenon is hypothesized to be a result of more favorable growth conditions at depth stimulating an increase in cyanobacterial biomass and potentially the biomass of other microorganisms. Specifically, groundwater entering the lake and/or increased heterotrophic respiration at depth may be significant contributors of limiting nutrients such as phosphorous to the system. This is supported by the specific conductance profiles and a general decrease in δ^13^C_DIC_ values with depth.

These results suggest that photoautotrophic metabolisms, and thus biological influences, are important in carbonate precipitation, at least at 26 m in Kelly Lake. It does not, however, rule out possible abiotic mechanisms contributing to microbialite formation in Kelly Lake. These findings are significant for understanding growth mechanisms of modern microbialite structures and the preservation of carbonate isotopic biosignatures in the geologic record. Furthermore, it is evident that some physical or chemical property of Kelly Lake is affecting the microbial growth at shallower depths. This discovery is important to comprehend factors that may limit microbial success and, ultimately, alter the pattern of microbialite growth and the preservation of isotopic biosignatures in both modern and ancient systems.

## Figures and Tables

**Table 1:** Quantitative PCR primers.

Primer	Product Size (Bp)	Annealing Temp (°C)	Reference
Eub338f ACTCCTACGGGAGGCAGCAG Eub518r ATTACCGCGGCTGCTGG Cya106f CGGACGGGTGAGTAACGCGTGA Cya359r * CCCATTGCGGAAAATTCCC	200	53	Fierer et al. [48]
	265	60	Nübel et al. [50]

* Custom Primer designed for this study.

**Table 2:** Physical properties measured for Kelly Lake surface and deep water, local groundwater wells, and Kelly Lake surface water inflow (Porcupine creek). N/A, data not available.

Sample	[DIC] (mg/L)	δ^13^C_DIC_ (‰)	pH	Conductivity (µs/cm)	[P] (µg/L)
Kelly surface water	36.5	−5.7 *^,‡^	8.41	326	2
Kelly deepwater (15 m)	N/A	−6.7 *	N/A	N/A	N/A
Kelly deepwater (30 m) ^†^	37.8	−7.2 *	8.28	334	4
Porcupine creek	40.7	−7.3	8.27	365	6
Jim well ^†^	41.7	−10.1	7.83	422	6
Guy and Ursula well ^†^	48.6	−10.1	7.71	626	7

* Taken from 2012 depth profile; ^†^ Reported in Lim et al. [37]; ^‡^ Average summer δ^13^C_DIC =_ −4.9 ± 1.1‰ (*n* = 8).

**Table 3:** Mean concentration of phospholipid fatty acids (PLFAs) (*n* = 3) relative to the dry weight and TOC of Kelly Lake microbialite surface material. Mean δ^13^C values for the carbonate and bulk organic material are also presented. Error values represent one standard deviation from the mean.

Sample Depth	[PLFAs] (µg/g Microbialite)	[PLFAs] (mg/g TOC)	δ^13^C_carb_ ‰ (PBD)	δ^13^C_org_ ‰ (PDB)
11 m	7.2 ± 4.4	0.4 ± 0.2	−2.7 ± 0.5	−29.8 ± 0.8
14 m	24.5 ± 11.0	0.8 ± 0.3	−3.8 ± 0.1	−27.6 ± 2.3
20 m	51.5 ± 12.7	1.3 ± 0.1	−3.8 ± 0.5	−30.8 ± 1.6
26 m	35.7 ± 15.6	1.4 ± 0.5	−0.3 ± 0.5	−34.2 ± 0.7

**Table 4:** PLFA distributions and δ^13^C values of representative microbialite samples at 11, 14, 20, and 26 m collection depths. δ^13^C values are reported as means ± one standard deviation from triplicate analysis. PLFA distributions are given in mol %.

	11 m		14 m		20 m		26 m	
PLFA I.D.	mol %	δ^13^C (‰)	mol %	δ^13^C (‰)	mol %	δ^13^C (‰)	mol %	δ^13^C (‰)
br14:0	0.8		1.5		0.3		0.5	
14:1	0.4		0.0		0.3		0.8	
14:0	6.0	−36.6 ± 0.5	5.4	− 43.7 ± 0.5	5.0	−44.3 ± 0.5	7.8	−48.3 ± 0.5
br15:0	0.0		0.0		0.0		0.4	
*i-*15:0	4.5	−32.5 ± 0.5	5.3	−32.7 ± 0.6	1.4	−34.2 ± 0.5	1.6	−35.0 ± 0.5
*a-*15:0	3.7	−35.1 ± 0.5	4.7	−31.8 ± 0.5	1.1	−35.0 ± 0.5	1.5	−34.0 ±0.5
15:0	1.4		1.2		0.4		0.5	
br16:0	2.1		1.3		0.0		0.0	
16:3	0.0		0.0		1.4		1.8	
16:2	0.0		0.0		2.1		2.0	
16:1	11.6	−42.0 ± 0.6	15.6	−39.4 ± 0.8	14.2	−41.6 ± 1.5	11.0	−44.1 ± 0.5
16:0	29.7	−38.0 ± 0.5	27.1	−42.5 ± 0.7	25.2	−42.1 ± 0.5	24.8	−46.8 ± 0.5
br17:1	0.0		0.6		0.5		0.2	
10me16:0	3.3		5.3		1.6		1.5	
*i-*17:0	1.5		0.2		0.4		0.1	
*a-*17:0	1.6		0.5		0.5		0.2	
17:1	0.0		0.0		0.4		0.2	
cy17:0	1.9		1.2		0.6		0.4	
17:0	1.2		0.0		0.3		0.1	
br18:0	0.0		0.0		0.2		0.0	
18:3	3.1		0.3	−42.9 ± 0.7*	2.9	−45.1 ± 0.6	1.8	−47.3 ± 0.5*
18:2	6.4		6.3		10.7	−45.7 ± 0.5	9.0	
18:1	15.1		17.2		21.1	−43.3 ±0.5	25.4	
18:0	3.1		1.5		1.4		0.8	
br19:0	1.2		0.5		0.4		0.4	
cy19:0	1.6		1.4	−33.8 ± 0.6	0.7		0.6	
20:4	0.0		0.8		1.5		1.7	−47.9 ± 0.6†
20:5	0.0		1.0		1.0		2.0	
20:3	0.0		0.0		0.6		0.7	
20:2	0.0		0.0		0.3		0.2	
20:1	0.0		0.6		1.3		0.9	
20:0	0.0		0.5		0.3		0.2	
22:6	0.0		0.0		0.2		0.0	
22:5	0.0		0.0		0.2		0.2	
22:1	0.0		0.0		0.4		0.0	
22:0	0.0		0.0		0.2		0.1	
24:1	0.0		0.0		0.2		0.0	
24:0	0.0		0.0		0.2		0.1	

* Value includes all unsaturated PLFA with a chain length of C18 (shown in grey); † Value includes all unsaturated PLFA with a chain length of C20 (shown in grey).
